# Characterizing the Conductivity and Enhancing the Piezoresistivity of Carbon Nanotube-Polymeric Thin Films

**DOI:** 10.3390/ma10070724

**Published:** 2017-06-29

**Authors:** Yingjun Zhao, Martin Schagerl, Christoph Viechtbauer, Kenneth J. Loh

**Affiliations:** 1 Christian Doppler Laboratory for Structural Strength Control of Lightweight Constructions, Johannes Kepler University Linz, 4040 Linz, Austria; martin.schagerl@jku.at (M.S.); christoph.viechtbauer@nemak.com (C.V.); 2 Institute of Structural Lightweight Design, Johannes Kepler University Linz, 4040 Linz, Austria; 3 Department of Structural Engineering, University of California, San Diego, 9500 Gilman Drive MC 0085, La Jolla, CA 92093-0085, USA; kenloh@eng.ucsd.edu

**Keywords:** carbon nanotube, lightweight design, nanocomposite, piezoresistivity, strain sensitivity, structural health monitoring

## Abstract

The concept of lightweight design is widely employed for designing and constructing aerospace structures that can sustain extreme loads while also being fuel-efficient. Popular lightweight materials such as aluminum alloy and fiber-reinforced polymers (FRPs) possess outstanding mechanical properties, but their structural integrity requires constant assessment to ensure structural safety. Next-generation structural health monitoring systems for aerospace structures should be lightweight and integrated with the structure itself. In this study, a multi-walled carbon nanotube (MWCNT)-based polymer paint was developed to detect distributed damage in lightweight structures. The thin film’s electromechanical properties were characterized via cyclic loading tests. Moreover, the thin film’s bulk conductivity was characterized by finite element modeling.

## 1. Introduction

The aerospace industry employs lightweight structural components that are robust against harsh operating conditions and simultaneously to attain higher fuel efficiencies. In addition to metals, fiber-reinforced polymer (FRP) composites are particularly favored by lightweight design due to their high strength-to-weight ratio and inertness to corrosion [1,2,3,4,5,6]. Many civil aircraft adopt FRPs to compose essential load-bearing parts, such as the control surfaces and wings. To further improve one’s fuel efficiency and to seek higher commercial profits, Airbus and Boeing even launched FRP-dominated aircraft (e.g., the A350 series and the 787 Dreamliner) whose entire fuselages are composed of carbon fiber-reinforced polymers (CFRPs) [3,4]. The Falcon 9 spacecraft from SpaceX also adopts a CFRP fairing to resist dynamic pressure during launching [7]. As one of the most expensive parts of a rocket, the fairing was able to be retrieved for future reuse, which can drastically reduce the manufacturing cost of a rocket [8].

These lightweight designed parts, however, should be closely monitored for their structural integrity to prevent unexpected structural failure, which may threaten life safety and cause severe property loss. Material defects, accumulated wear-and-tear, and other structural damage features should be identified at the earliest possible stage for reducing the costs of delayed rehabilitation and maintenance. As a result, non-destructive evaluation (NDE) and structural health monitoring (SHM) technologies can enhance the safety and performance of structural systems through periodic and in situ inspections and condition assessment.

Several popular NDE methods are currently used for schedule-based inspections of aircraft, such as visual inspection [9], ultrasonic testing [10,11], and X-ray scanning [12,13], to name a few. Visual inspection remains the most popular way of identifying visible surface damages [9]. Ultrasonic transducers are effective tools for localizing damage in metallic structures. An acoustic wave sent from an actuator transmitting through the thickness of a structure will reflect due to the presence of material discontinuities or interfaces between different materials, thereby enabling defects, voids, and cracks to be captured along its traveling path. Under scanning mode, the technique can effectively identify surface and subsurface damage features over the scanned area. Radiography methods detect minor thickness changes caused by material defects or cracks, localizing material-level imperfections with high precision and accuracy. These techniques are also demonstrated to be effective at locating, quantifying, and qualifying damage in FRP structures [10,11,12]. However, the majority of these techniques are offline methods that can only be performed periodically, especially since they are extremely labor-, time-, and cost-intensive. On the other hand, online assessment of a structure reveals in situ performance metrics during operational loading conditions while enabling real-time monitoring of damage initiation and propagation. Strain gages, fiber-optic sensors, acoustic emission transducers, and other more advanced sensors, such as Comparative Vacuum Monitoring sensors, can be installed in a network to observe real-time structural performance [9,14,15,16]. However, some of these sensors cannot be used in harsh operating environments. Moreover, for need of higher spatial sensing resolution, a densely distributed sensor instrumentation may adversely impact the structure’s self-weight and cost [16]. To overcome these challenges, the lightweight design research community is motivated to develop a sensor system that specifically addresses these challenges. The next generation SHM system for aerospace structures needs to be reliable, lightweight, integrated with a structure during manufacturing, and be able to perform in situ structural monitoring over a large, spatially distributed region [9].

To fill this gap, an emerging and viable approach is to assemble smart materials using unique materials and bottom-up fabrication methods offered by the nanotechnology domain [17]; for instance, carbon nanotubes (CNTs) are 1D nanomaterials with outstanding mechanical strength and electrical conductivity. Individual tubes can be thermally and electrically conductive like metals, reaching up to 1 TPa in terms of their Young’s modulus [18]. They can be assembled in appropriate polymer matrices to form nanocomposites with high ductility and electrical conductivity. In addition, these CNT–polymer nanocomposites are widely investigated for fabricating flexible sensors that respond to strain, pressure, and presence of oxygen [18,19,20,21,22], to name just a few. Lipomi et al. [19] fabricated transparent CNT–poly(dimethylsiloxane) (PDMS) thin films via spin-coating; these thin sheets can sustain large strains while exhibiting predictable changes in resistance and capacitance responses. Park et al. [20] fabricated strain-sensitive thin films using multi-walled carbon nanotubes (MWCNT) and polyethylene oxide (PEO) solutions; it was observed that the thin film’s resistance response varied in both linear and nonlinear manners. Liu et al. [23] micropatterned MWCNT-PDMS strain gages via a laser beam, and the nanocomposite was stamp-transferred onto a PDMS substrate. The fabricated gage responded to strains larger than 45%. Other studies investigated sensors that could withstand large strains by using different nanofillers. Lin et al. [24] assembled a graphene–elastomer composite via hot compression with a very low filler content to reach the percolation threshold. The sensor was characterized by a gage factor of 82.5 and withstood strains over 100%. Wei et al. [25] embedded silver nanowires within waterborne polyurethane to screen-print a bending sensor whose electrical response remained highly repeatable after thousands of loading cycles. Loh et al. [26] assembled CNT–polyelectrolyte thin films via layer-by-layer (LbL) method, which is a technique that employs van der Waal attraction between oppositely charged materials to realize self-assembly; their characterized strain sensitivity or the gage factor was 6.5. Despite these advances, many of these nanomaterial-based sensors require complex and time-consuming fabrication methods, and the size and shape of thin films fabricated are strictly limited by the dimensions of the substrate used.

Alternative techniques are needed to rapidly assemble thin films that conform to complex structures with enhanced scalability. Amjadi et al. [27] sandwiched MWCNT–Ecoflex nanocomposites between layers of Ecoflex as a strain sensor by screen printing. A strong binding between the nanoparticles and the matrix was observed as the stress–strain hysteresis loop shrunk. Goncalves et al. [28] screen-printed MWCNT–poly (vinyl alcohol) strain sensors with a gage factor of up to 3. Due to its excellent flexibility, poly(vinylidene fluoride) (PVDF) was chosen as the matrix material for fabricating strain sensors with a reported gage factor of 6.2 [29]; moreover, Loyola et al. [30] and Mortensen et al. [22] introduced an airbrushing technique to instantly spray PVDF-MWCNT paint onto various structural surfaces, which provided a cost-effective assembling method for the aerospace industry. This nearly weightless layer of paint, without extra molding or cover, could act as a damage sensing layer that is able to identify the severity and locations of impact damage when combined with an electrical impedance tomography algorithm. However, its strain sensitivity was found to be lower than many of the aforementioned MWCNT-based strain sensors and is not sufficient at capturing mild deformations such as strains less than 5% [31].

Therefore, the goal of this study is to design an MWCNT-based strain sensor with enhanced electromechanical properties that could still be fabricated in a scalable fashion. The first part of this study was dedicated to enhancing the strain sensitivity of MWCNT thin films by replacing polystyrene sulfonate (PSS) in the original ink solution with Pluronic [22,30]. Pluronic is a triblock-copolymer that dissolves in both water and oil; due to its unique hydrophilic and hydrophobic characteristics, it is well-known for stabilizing nanoparticles in aqueous solutions for extended periods of time [32,33]. To compare their strain sensing properties, both MWCNT-Pluronic-PVDF and MWCNT-PSS-PVDF thin films were sprayed onto polyamide coupons, which were subjected to tensile loads to characterize their strain sensitivities. Tensile cyclic load tests were performed to further examine their stability and repeatability. The second part of this study was to characterize their scalability. The electrical properties along the gage length of an MWCNT-PSS-PVDF thin film were characterized as its width was varied. A square-shaped thin film was fabricated with two electrodes attached onto both its ends, while two-point probe resistance measurements were measured each time after a partial width of the film was removed. The resistance change with respect to the width change was plotted, and a finite element analysis (FEA) model adopted the data and thereafter simulated how additional changes in geometry affected film properties.

## 2. Experimental Details

### 2.1. Thin Film Fabrication

Essential thin film fabrication materials such as poly(sodium 4-styrenesulfonate) (PSS), Pluronic^®^ F-127, and MWCNTs were all purchased from Sigma-Aldrich Corporation (St. Louis, MO, USA). The MWCNT-PSS-PVDF paint was prepared in accordance to the instruction mentioned in Loyola et al. [30] and Mortensen et al. [22]. A 2 wt % PSS solution was prepared by subjecting it to 60 min of bath sonication and until no visible particles were seen. The MWCNT powder was added to the solution at approximately 10 wt %, and the mixture was subjected to a 0.5 s-on, 0.5 s-off tip sonication cycle for 60 min (Bandelin Sonopuls GM 70, 70 W, 20 kHz, Bandelin electronic GmbH & Co. KG, Berlin, Germany). A small amount of N-methyl-2-pyrrolidinone (NMP) was added during sonication to improve adhesion between polymer chains and individual nanotubes. Additional sonication time could be added if the final solution does not appear homogeneous, which is the ink’s expected state. A sample of PVDF latex was received from Arkema Inc. (Kynar Aquatec ARC, Arkema, Colombes, France); it was mixed via stirring with the MWCNT-PSS ink to increase its viscosity for an easier spray. PVDF is a good material binder that helps enhance the adhesion between the ink and the surface to be sprayed. PVDF was added at such an amount that the MWCNT weight content of the final dry thin film was maintained at 5 wt %. The MWCNT-Pluronic-PVDF paint was prepared in a similar fashion. 2 wt % Pluronic solution was prepared by stirring the mixture at 50 °C until no visible polymer particles could be seen. It was observed that the shelf life of the MWCNT-Pluronic ink is much longer than that of the MWCNT-PSS ink (e.g., several weeks versus several days), possibly due to Pluronic’s outstanding ability at stabilizing nanoparticles.

**Figure 1:**
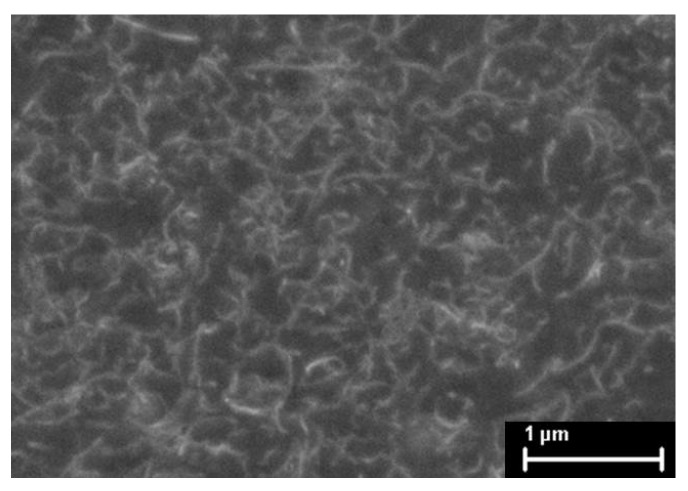
The SEM image shows the percolated morphology of the MWCNT-Pluronic-PVDF thin film.

Each paint was sprayed over the middle section of a polyamide 6 (PA 6) tensile test coupon (180 mm × 40 mm). The MWCNT-PSS-PVDF paint was loaded into an airbrush (0.5 mm tip, Agora-Tec GMBH, Schmalkalden, Germany) and sprayed over a PA 6 coupon surface, which was masked to leave a 20 mm × 60 mm window for thin film attachment. To paint the MWCNT-Pluronic-PVDF thin film, an infrared lamp (Philips IR 250 RH, 250 W, Amsterdam, The Netherlands) was hung over the PA 6 coupon so as to maintain the coupon’s surface temperature at approximately 55 °C. Due to its low viscosity, the MWCNT-Pluronic-PVDF paint had to be quickly thickened as it adhered to the bonding surface via elevated temperature and lowered humidity [34]. Figure 1 shows the scanning electron microscope (SEM) image of the MWCNT-Pluronic-PVDF thin film. The morphology of the thin film consists of uniformly distributed MWCNTs that ensures uniform electrical properties throughout the entire nanocomposite. For each kind of paint, five specimens were prepared, which were left to air dry for at least 1 h after being sprayed.

**Figure 2:**
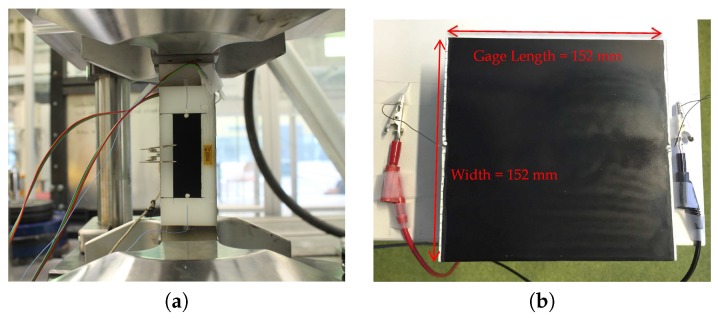
(**a**) A painted PA 6 coupon was mounted in the load frame prior to testing; (**b**) An MWCNT-PSS thin film is sprayed over a G10-FR4 plate. Two wires were attached for measuring its gage resistance response with respect to width changes (starting with 152 mm).

Two electrodes were installed, each at the midpoint of the thin film’s width, as seen in Figure 2a. An electrical wire was fixed at each electrode by using a two-part silver epoxy (Silver Conductive Epoxy, EPO-TEK H20E, Epoxy technology, Inc., MA, USA) that cured at 80 °C for 3 h. The silver epoxy has a low sheet resistance of 16 Ω/sq/mil that ensures low contact resistance of each electrode. Compared to the intrinsic resistance of each thin film (i.e., 600–700 Ω), contact resistance of the electrodes can be considered negligible. Moreover, the wires were tightly fixed to the specimen due to the epoxy component; therefore effects from electrode movement can be omitted from consideration. As a result, all the resistance measurements in this study were taken by a two-point resistance probe.

### 2.2. Quasi-Static Cyclic Tensile Test

All the PA 6 coupons were subjected to a quasi-static uniaxial tensile test with a cyclic loading pattern. As demonstrated in Figure 2a, each coupon was mounted in an MTS 647 hydraulic wedge grip (MTS Systems Corporation, Eden Prairie, MN, USA) with an initial grip separation distance of 135 mm. To measure the strain response, a strain gage was attached next to the thin film in the middle of the gage length of the coupon. Both the strain gage data and the two-point probe resistance measurement data of the thin film were collected using an HBM QuantumX MX840B multichannel data acquisition system (HBM Germany, Darmstadt, Germany) at a sampling rate of 100 Hz. Each coupon was loaded using a linear saw-tooth pattern with a loading peak of 0.8 kN for five runs, followed by 1.6 kN for another five runs. During the test, an extensometer was also installed on the back of the coupon; it detected slight bending for almost every loaded coupon (e.g., strain values calculated from the extensometer were always 100–200 μm higher than that from the strain gage reading). This effect was considered to be minor; therefore, the result was omitted from the following discussion.

### 2.3. Two-Point Probe Resistance Measurements

In this test, two-point probe resistance measurement of a rectangular thin film was performed each time after its width was reduced. A 152 mm × 152 mm flame-retardant garolite (G10-FR4) composite plate was sprayed with the MWCNT-PSS-PVDF paint. An electrode was attached at the midpoint of the thin film’s width, and another electrode was attached on the opposite edge, as shown in Figure 2b. The resistance measurement was taken using an HP 34401A multimeter (Keysight Technologies, Santa Rosa, CA, USA). After taking a measurement, the thin film’s width was reduced by slicing off 5 mm the thin film along its gage length, alternating from one side to the other, followed by a resistance measurement after every reduction. When the thin film’s width reached 7 mm, the reduction step was reduced to 1 mm, until a thin film of only 1 mm in width was left. To further investigate how the thin film’s length change may impact the result, the resistance measurement was adopted by a FEA software to model the testing scheme. Using the same software with an estimated conductivity value, the aforementioned test was simulated with various thin film lengths. A MATLAB-based open-source software EIDORS was used to perform the simulation. This modeling software can simulate the 2D and 3D electrical behavior of a conductor using the finite element approach [35].

## 3. Results and Discussion

### 3.1. Piezoresistivity Characterization

**Figure 3:**
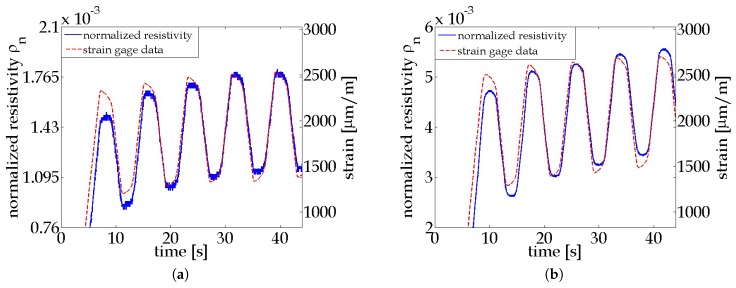
Time history plots of a PA 6 coupon with a (**a**) MWCNT-PSS-PVDF thin film and a (**b**) MWCNT-Pluronic-PVDF thin film. The normalized resistivity patterns overlap well with their corresponding strain profiles applied on each coupon.

To characterize and compare the strain sensitivity of each material, the resistance measurement collected in Section 2.2 should be normalized with respect to the thin film’s dimension. The bulk resistivity, which measures the dimensionless electrical property, can be calculated as

(1) $$\rho =\frac{R\;A}{L}$$

where ρ stands for resistivity, *R* is the resistance measured along the gage length *L*, and *A* is the cross-sectional area along *L*. The normalized resistivity, $\rho_{n}$, can therefore be calculated as

(2) $$\rho_{n}=\frac{\rho -\rho_{0}}{\rho_{0}}$$

where $\rho_{0}$ is the initial resistivity of the film in its pristine condition. Figure 3a,b plot the time history of strain and normalized resistivity of MWCNT-PSS-PVDF and MWCNT-Pluronic-PVDF thin films, respectively. It can be observed that the normalized resistivity profile of each thin film overlaps well with its own strain profile, and this consistency is maintained even after multiple loading cycles. This result shows that both thin films are sensitive to strains, and their electrical behavior can be correlated to a specific strain state in a repeatable manner.

**Figure 4:**
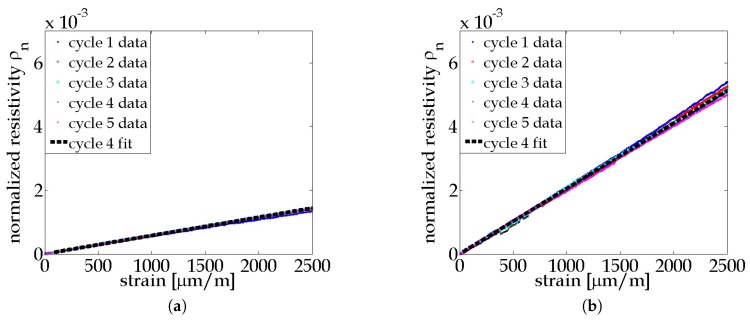
Plots of normalized resistivity of the (**a**) MWCNT-PSS-PVDF thin film and the (**b**) MWCNT-Pluronic-PVDF thin film with respect to the strain responses of their PA 6 coupons.

It is noted that the strain patterns in Figure 3a,b fall in approximately the same range, but the $\rho_{n}$ responses of MWCNT-Pluronic-PVDF thin films vary to a larger extent than that of the MWCNT-PSS-PVDF thin film. This indicates that the former nanocomposite exhibits a higher strain sensitivity than the latter. The strain sensitivity, or the gage factor of the piezoresistive specimen, can be calculated as

(3) $$GF=\frac{\rho_{n}}{\varepsilon }$$

where ε is applied strain along the gage length. Figure 4a,b plot the normalized resistivity of each loading cycle against their corresponding strain gage data for all the coupons. A linear trend is observed in both plots, indicating a linear correlation between resistivity and strain for both types of thin films. A linear least-squares regression fit for each cycle of the $\rho_{n}$ vsersus ε data was performed, and the average values per coupon type were calculated to be the gage factor of each type of thin film. The calculated gage factor of MWCNT-PSS-PVDF thin film is 0.62 ± 0.12, which is similar to the result reported in Mortensen et al. (e.g., 0.72 ± 0.02) of free-standing MWCNT-PSS-PVDF thin films [22]. The gage factor of the MWCNT-Pluronic-PVDF thin films is 2.06 ± 0.18, which is approximately three times that of the MWCNT-PSS-PVDF thin films and close to that of a foil-typed strain gage. This may be due to the more stable dispersion of CNTs in Pluronic solution. It is clear that Pluronic as a polymer stabilizer outperforms PSS for enhanced strain sensitivity of an MWCNT-based strain sensor; the hypothesis is that, compared to PSS, the nanotube stabilizing mechanism of Pluronic improves the tunneling effect among individual tubes, thereby resulting in a larger change in the thin film’s bulk electrical response in response to strain. As a result, the MWCNT-Pluronic-PVDF thin film can be used to capture mild strains and deformation. It should be noted that the geometric effect due to the Poisson’s effect of the thin film during loading is not accounted for in Equation (3). While the Poisson’s ratio of the thin films remains unknown, it is true that the geometric effect can affect the strain sensitivity of a foil-typed strain gage. Therefore, to compare the thin film’s strain sensing performance to that of strain gages, the result from Equation (3) is adequate. Moreover, the Poisson’s effect in real-world applications would be dominated by that of the material of the structure to which the sensor is attached, rather than by the thin film strain sensor itself; therefore, the impact of geometric effects on the thin film’s strain sensitivity is not discussed in this study.

**Figure 5:**
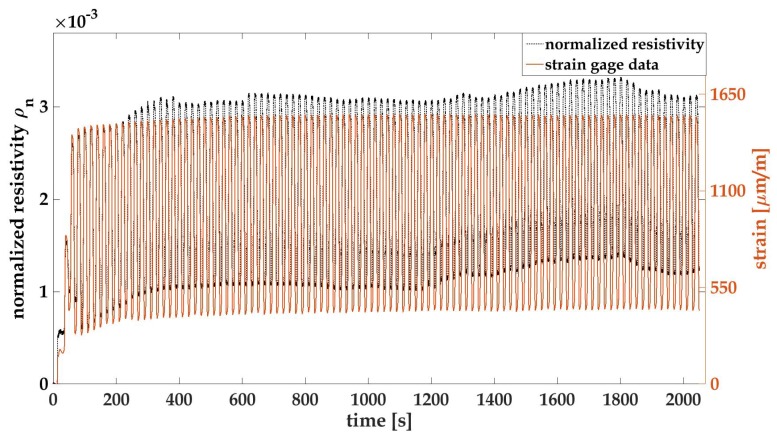
A 100-cycle load–unload tensile test was carried out on the specimens with MWCNT-Pluronic-PVDF thin films.

**Figure 6:**
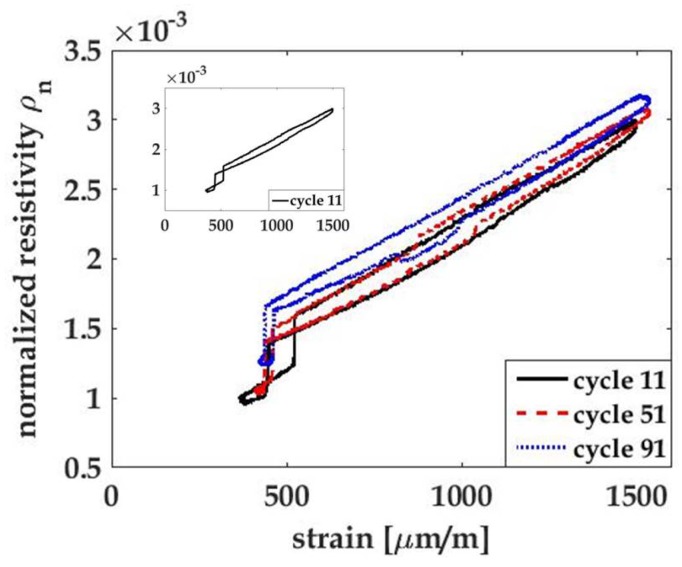
The hysteresis plot of the MWCNT-Pluronic-PVDF thin film was acquired from the 100-cycle test.

It is observed in both Figure 3a,b that both strain gage signals and the thin film’s resistance data drift upwards as the number of loading cycles increases. In order to further observe the stability of both signals, load–unload cyclic tensile tests were conducted on specimens with MWCNT-Pluronic-PVDF thin films. Figure 5 shows one of the testing results. It should be noted that cyclic load was applied at a constant load rate of 0.1 kN/s, and the peak-to-peak values were 0.1 to 1 kN. The reason that the unloading cycle did not unload to 0 kN was that it was difficult for the load frame’s hydraulic pump to reach 0 load, and 0.1 kN was the lowest force value that the testing equipment could attain. This partially explains why both the strain gage and the thin film’s resistance did not return to their original values after each loading cycle. Similar to results in Figure 3b, the strain gage signal shown in Figure 5 drifts upwards during the first 10 loading cycles and that of the thin film’s resistance shows a similar pattern. This is due to the viscoelastic behavior of the testing coupon material, PA 6, for which an instantaneous softening of applied stress takes place immediately after the material is loaded. Therefore, when the applied load returned to the original minimum value, material elongation could not be totally recovered and therefore stayed at a slightly higher value [23]. This phenomenon vanished after nearly 10 cycles, as indicated by the strain gage data. The thin film results, however, show a random drifting pattern till the end of the test profile, indicating an unsteady electromechanical behavior. Many reasons may contribute to the drifting signal of the thin film. First, the thin film is a PVDF-based nanocomposite that exhibits viscoelastic behavior. Due to the Payne effect, which is a unique property of elastomers with fillers under small strain magnitude, a loss of the storage modulus of the thin film starts from the first cycle [36,37]. This is mainly due to the rearrangement of the MWCNT network responding to applied cyclic loads, and the electrical signals thus fluctuate every time when a new electrical path is formed [24,36]. Moreover, due to the load-controlled nature of the experiment, the strain rate of the specimen near both peaks could vary drastically, causing different degrees of stress relaxation of the thin film to take place and invoking random electrical behavior [38]. However, the gage factor of the thin film is not influenced throughout the cyclic loads [28]. The hysteresis analysis shows similar results as illustrated in Figure 6. The hysteresis loop of the thin film stays nearly intact in different cyclic periods, indicating reversible material behavior. Moreover, the narrow band of the loop indicates strong bonding between MWCNTs and the polymer matrix [27]. The jump of the thin film’s resistance observed in Figure 6 is due to the jump of the clamping device under low loads to overcome its self-weight; a similar signal was also observed in the grip separation data.

It should be pointed out that the entire test was performed in constant room temperature; the samples were left in the same room for at least one day before testing. Temperature is also an important factor determining a polymeric nanocomposite’s viscoelastic behavior [23,27,38], therefore future studies that characterize the thin film’s electromechanical performance under temperature influence will be carried out.

### 3.2. Characterization of MWCNT-PSS-PVDF Thin Film’s Conductivity

**Figure 7:**
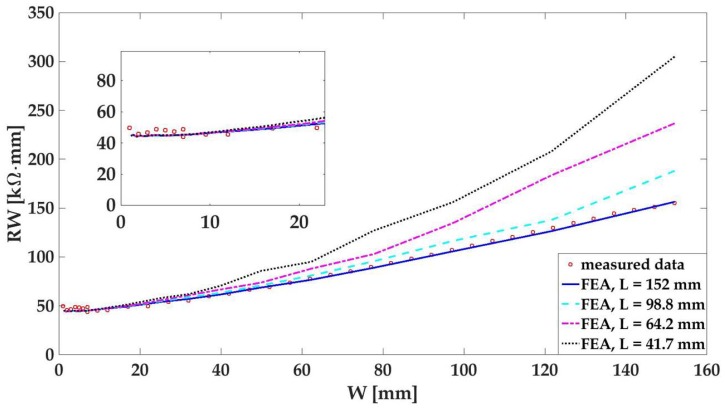
The plot of the gage resistance multiplied by the width of a MWCNT-PSS-PVDF thin film was simulated by EIDORS.

Figure 7 illustrates the measurement data from the two-point probe resistance measurement with respect to width variations. Since the thin film’s length and thickness remained unchanged throughout the experiment, it is equivalent to plot the resistance multiplied by its width, or RW, to represent the thin film’s equivalent resistivity, according to Equation (1). The measurement data suggests that the equivalent resistivity of the thin film stays intact until *W* became larger than 10 mm. As a material property, it can be concluded that Equation (1) remains a valid expression for calculating the thin film’s resistivity, and the nanocomposite behaves like a 1-D conductive wire. When *W* exceeds 10 mm, RW becomes width-dependent, indicating that Equation (1) is no longer valid for resistivity calculation. At this point, it can be concluded that not much material outside the width of 10 mm is effective in carrying the electrical current passing through the dedicated gage length. In other words, the thin film may not capture any strain deformations or damage that are more than 10 mm away in the transverse direction of the gage path using a two-point probe measurement.

The solid blue curve in Figure 7 is a data-fitting curve derived from the FEA model [35]. There is good agreement with the simulated data to the measurement data, when the bulk conductivity of the thin film was set to 140 S/m with an electrode size of 6 mm. Using the same input parameters, more simulations were performed with different thin film lengths. The dotted curves in Figure 7 plot the simulated results of thin films with different gage lengths at 98.8 mm, 64.2 mm, and 41.7 mm. As the gage length decreases, minor shifts of the thin film’s stabilizing point are detected, i.e., when the length is reduced by nearly 73% percent (from 152 mm to 41.7 mm), the RW value does not drift up until its width reaches approximately 10 mm. This result indicates that the scalability of the polymeric CNT thin film is dominated by its intrinsic electrical property and the electrode size. It shows that the current path in the transverse direction of the thin film is constrained. This knowledge is essential for future applications of the thin film being painted over a large distributed region so that the optimized electrode size and electrode spacing can be determined.

## 4. Conclusions

This study aims to enhance the strain sensitivity and scalability of an MWCNT-based polymer paint by using Pluronic as the nanoparticle stabilizer. In this study, several MWCNT–polymeric thin films were rapidly assembled using a compressed-air spraying technique. The strain sensitivity was characterized via cyclic tensile tests on coupons with thin films directly painted as strain sensors. By replacing the stabilizing polymer in the original paint formula with Pluronic, the strain sensitivity of the MWCNT-embedded thin film was improved by more than three times to a gage factor of 2.06 ± 0.18, which is close to a foil type strain gage. The second part of this study characterized the scalability of a rectangular-shaped MWCNT-PSS-PVDF thin film in terms of its electrical behavior. By conducting a simple two-point probe resistance measurement across the thin film’s fixed gage length of 152 mm, it was observed that its electrical behavior would not be significantly influenced by its transverse direction dimension when it is wider than or equal to approximately 10 mm. An FEA model simulated the testing scheme with different gage lengths, confirming that this influence in transverse dimension mostly comes from the material’s intrinsic electrical properties and the electrode size. Future research will focus on applications of MWCNT-Pluronic-PVDF thin films applied onto real-world structures as a spatial damage sensor. Other possible fabrication methods such as inkjet-printing will also be considered. The results from this study have provided adequate knowledge for the application of carbon nanotube-based thin films as strain sensors, including their future application on lightweight structures in the real-world.
